# Temporal changes in the radiocesium distribution in forests over the five years after the Fukushima Daiichi Nuclear Power Plant accident

**DOI:** 10.1038/s41598-017-08261-x

**Published:** 2017-08-15

**Authors:** Naohiro Imamura, Masabumi Komatsu, Shinta Ohashi, Shoji Hashimoto, Takuya Kajimoto, Shinji Kaneko, Tsutomu Takano

**Affiliations:** 1 Center for Forest Restoration and Radioecology, Forestry and Forest Products Research Institute, Tsukuba, Ibaraki 305-8687 Japan; 2 Department of Mushroom Science and Forest Microbiology, Forestry and Forest Products Research Institute, Tsukuba, Ibaraki 305-8687 Japan; 3 Department of Wood Properties and Processing, Forestry and Forest Products Research Institute, Tsukuba, Ibaraki 305-8687 Japan; 4 Department of Forest Soils, Forestry and Forest Products Research Institute, Tsukuba, Ibaraki 305-8687 Japan; 5 Tohoku Research Center, Forestry and Forest Products Research Institute, Morioka, Iwate 020-0123 Japan; 6 Research Planning and Coordination Department, Forestry and Forest Products Research Institute, Tsukuba, Ibaraki 305-8687 Japan

## Abstract

To elucidate the temporal changes in the radiocesium distribution in forests contaminated by the Fukushima Daiichi Nuclear Power Plant accident, we monitored the ^137^Cs concentration and inventory within forests from 2011 to 2015 across nine plots containing variable tree species and different contamination levels. The ^137^Cs concentrations in needles and branches decreased exponentially at all coniferous plots, with effective ecological half-lives of 0.45–1.55 yr for needles and 0.83–1.69 yr for branches. By contrast, the ^137^Cs concentration in deciduous konara oak leaves did not change over the five years. The concentration of ^137^Cs in oak wood increased by 37–75%, whereas that in Japanese red pine decreased by 63% over the five years. In Japanese cedar and hinoki cypress, the ^137^Cs concentration in wood showed an increasing trend in half of the plots. The changes in ^137^Cs in the organic and mineral soil layers were not strongly related to the tree species or contamination level. Our multi-site, multi-species monitoring results revealed that the pattern of temporal changes in radiocesium in the 9 forest plots was similar overall; however, changes in ^137^Cs in needles/leaves and wood differed among tree species.

## Introduction

After the Fukushima Daiichi Nuclear Power Plant (FDNPP) accident, a large volume of radionuclides was released into the atmosphere. Consequently, serious radioactive fallout resulted over a broad area of northeastern Japan^1, 2^, which is mostly covered by forests (ca. 70%). Radiocesium isotopes (^137^Cs and ^134^Cs) with long physical half-lives, particularly ^137^Cs (30 years), represented a major portion of the radionuclides released as a result of the accident. Therefore, prolonged ^137^Cs contamination in these forest ecosystems is a major concern^3^.

Such forest contamination has resulted in a decline in timber production and a variety of additional problems, including the contamination of edible forest products, such as wild mushrooms, plants, and animals, and risks of internal and external exposure to forest workers and local residents. To facilitate the application of countermeasures and to evaluate the risks of these exposures, monitoring the temporal changes in the ^137^Cs concentration and inventory in contaminated forests is essential. In Fukushima and the surrounding prefectures of Ibaraki and Tochigi, approximately 30% of the forests are dedicated to coniferous plantation for timber production, with Japanese cedar (*Cryptomeria japonica*) and hinoki cypress (*Chamaecyparis obtusa*) as the two main plantation species^4^. Secondary forests are also commonly found in this area, where deciduous broad-leaved (DBL) species such as konara oak (*Quercus serrata*) predominate. Japanese red pine (*Pinus densiflora*) is often found with broad-leaved trees in mixed forests. Due to the presence of such diverse forest types and terrain characteristics, temporal changes in the ^137^Cs distribution within forests may differ across sites. In addition to the various forest types found in these areas, an airborne survey revealed that contamination levels vary between locations by several orders of magnitude^5^. Although the influence of the difference in contamination level to ^137^Cs behaviour in forests would be negligibly small^6^, it would be beneficial to confirm the similarity in ^137^Cs behaviour in forests with different contamination levels. Therefore, monitoring the temporal changes in ^137^Cs at multiple plots covering various forest types and contamination levels is important to document the overall temporal changes in the ^137^Cs concentration and inventory across broad areas of contaminated forest.

**Figure 1:**
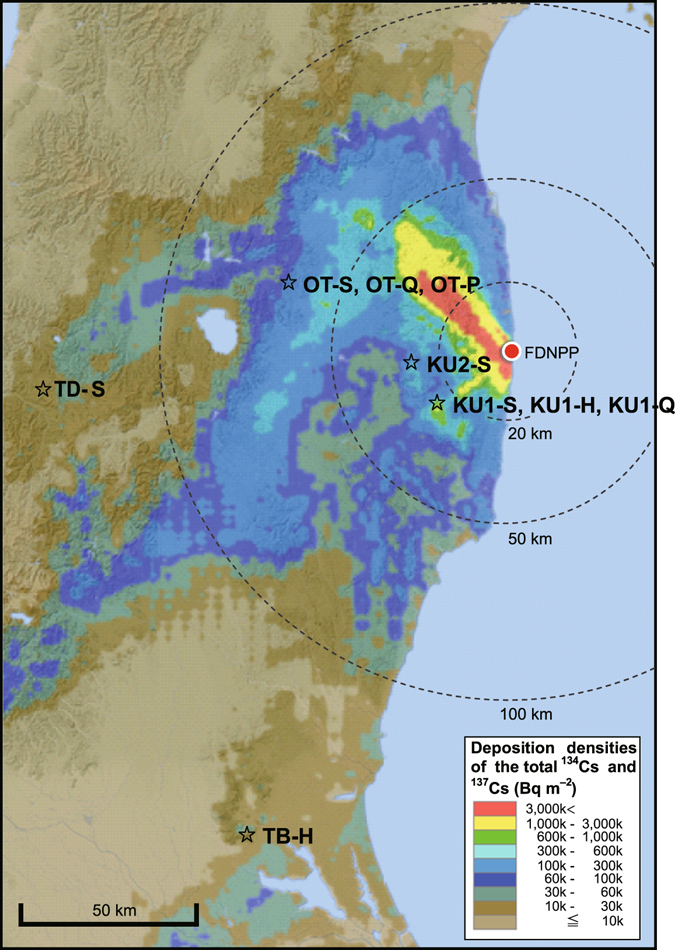
Locations of the study plots. The study plots are shown in a distribution map of ^137^Cs and ^134^Cs deposition levels on June 28, 2012 that was generated using the website “Extension Site of Distribution Map of Radiation Dose, etc”. prepared by MEXT, Japan^31^. The Extension Site of Distribution Map of Radiation Dose, etc., was created by editing GSI Tiles (elevation)^39^. KU1 and KU2: Kawauchi sites, OT: Otama site, TD: Tadami site, TB: Mt. Tsukuba site; -S: Japanese cedar plot; -H: hinoki cypress plot; -Q: DBL species-dominant plot; -P: Japanese red pine-dominant plot.

We began monitoring the radiocesium (^137^Cs and ^134^Cs) contamination in trees and soils in August 2011 (half a year after the accident) in five forest plots that differed in terms of the contamination level and dominant species (Table 1 and Fig. 1). The monitoring plots included three Japanese cedar plantations in Kawauchi Village (26 km from the FDNPP), Otama Village (66 km), and Tadami Town (134 km) (referred to as KU1-S, OT-S, and TD-S, respectively) and two mixed forest plots adjacent to OT-S that were dominated by broad-leaved trees (OT-Q) or Japanese red pine (OT-P). In these plots, we monitored the concentrations and inventories of radiocesium in needles/leaves, branches, bark, wood, soil surface organic layer, and mineral soil layers. In our previous paper, we reported the ^137^Cs distributions in forests observed in the summer of 2011, representing the phase immediately following the contamination^7^. The first year of observations revealed that the ratio of aboveground ^137^Cs (needles/leaves, branches, bark, and wood) to the total amount of ^137^Cs deposited in the forests varied from 18 to 52% across the various sites. Belowground, most of the ^137^Cs was distributed across the organic layer (16–49%) and along the mineral soil surfaces (20–44%) in 2011. Radiocesium was also detected in the internal tissues of trees (sapwood and heartwood), which had not been exposed to deposition, although these concentrations were much lower than those in the external tissues (needles/leaves, branches, and bark).

**Table 1:** General characteristics, biomass of aboveground components, and forest floor surface densities of belowground components at the study plots.

Plot	KU1-S	KU1-H	KU1-Q	KU2-S	OT-S	OT-Q	OT-P	TD-S	TB-H
Latitude	37°17′18″	37°17′22″	37°17′22″	37°22′53″	37°34′40″	37°34′15″	37°34′14″	37°19′28″	36°10′22″
Longitude	140°47′48″	140°47′33″	140°47′30″	140°42′58″	140°18′20″	140°18′29″	140°18′30″	139°31′15″	140°10′33″
Distance from FDNPP (km)	26	27	27	28	66	66	66	134	160
Elevation (m)	660	720	720	690	730	760	750	790	360
Topography	Flat and steep	Flat	Flat	Flat	Flat and steep	Flat	Flat	Steep	Steep
Air dose rate at 1 m height (μSv h^−1^)^a^	3.05 ± 0.42	3.70 ± 0.22	3.16 ± 0.09	1.29 ± 0.39	0.31 ± 0.02	0.33 ± 0.02	0.33 ± 0.02	0.12 ± 0.02	0.16 ± 0.02
^137^Cs deposition (kBq m^−2^)^b^	630	470	470	160	41	44	44	10	33
Dominant sp.	*Cryptomeria japonica*	*Chamaecyparis obtusa*	*Quercus serrata*	*Cryptomeria japonica*	*Cryptomeria japonica*	*Quercus serrata*	*Pinus densiflora*	*Cryptomeria japonica*	*Chamaecyparis obtusa*
Stand age (yr)	43	26	26	57	42	43	43	38	43
Density (ha^−1^)^c^	975/569	1330/−	−/1750	733/225	1117/−	550/654	938/375	1105/133	2133/−
Mean DBH (cm)^c^	18.8/14.3	17.6/−	−/13.1	30.9/25.8	24.8/−	19.0/17.5	18.8/15.6	19.9/17.2	20.6/−
Aboveground (kg m^−2^)^c^									
Needles/leaves	1.2–1.4/0.2	0.9–1.2 /−	−/0.3	3.0–3.3/0.00	2.3–2.7/−	0.3/0.3	0.4–0.5/0.1–0.2	1.5–1.8/0.1	2.3–2.5/−
Branches	0.7–0.9/0.8–0.9	1.1–1.6/−	−/1.2	2.6–2.9/0.02	1.5–1.8/−	0.7/1.3–1.5	1.0–1.1/0.6–0.7	0.9–1.1/0.3–0.4	3.2–3.6/−
Bark	0.4–0.5/0.7	0.6–0.8/−	−/1.1	1.0–1.5/0.01–0.02	0.9–1.0/−	0.3/0.6–0.7	0.4/0.3–0.4	0.7–0.8/0.2	1.0–1.6/−
Wood	6.6–8.1/3.3–3.7	9.9–12.7/−	−/6.0	19.7–21.0/0.1	13.7–16.6/−	4.4–4.8/4.2–4.8	6.6–6.9/2.1–2.5	8.3–10.8/0.9–1.3	23.6–26.7/−
Tree total	8.9–10.8/5.0–5.5	12.5–16.4/−	−/8.6	26.2–28.7/0.1–0.2	18.4–22.0/−	5.7–6.1/6.4–7.2	8.4–8.8/3.2–3.7	11.3–14.4/1.4–1.9	30.2–34.3/−
Belowground (kg m^−2^)									
Organic layer	1.1–1.6	1.1–1.5	1.0–1.2	1.4–2.8	1.6–2.1	0.9–1.5	1.0–1.7	1.1–1.6	0.8–1.5
Mineral soil 0–5 cm	15.7–19.6	14.4–17.2	18.0–19.4	10.8–14.6	11.9–14.3	13.9–17.9	16.2–18.7	15.3–21.3	16.5–17.4
Mineral soil 5–10 cm	22.8–29.6	22.8–23.8	23.4–26.2	14.6–22.6	17.6–19.0	24.2–25.8	23.7–28.7	21.8–33.1	24.1–26.6
Mineral soil 10–15 cm	27.1–32.7	22.6–27.3	27.9–29.7	22.5–28.3	19.9–22.4	26.4–30.8	25.1–28.8	27.6–47.4	25.6–27.4
Mineral soil 15–20 cm	29.5–36.3	24.7–27.8	30.1–33.8	23.8–29.3	20.5–25.0	28.3–31.6	24.3–32.2	33.7–40.9	23.5–27.6
Sampling date									
2011	31 Aug	^—^	^—^	28 Nov	9, 11 Aug	9–10 Aug	9–10 Aug	7–8 Sep	16 Feb, 2012
2012	23, 27 Aug	24, 28 Aug	24, 28 Aug	20 Sep	1, 6 Aug	2, 7 Aug	2, 7 Aug	3–4 Sep	28 Jan, 5 Feb, 2013
2013	26 Aug	27 Aug	27 Aug	27–28 Aug	1, 5 Aug	2, 6 Aug	1–2, 6 Aug	4, 10–11 Sep	25 Sep, 3 Oct
2014	25–26 Aug, 3 Sep	26 Aug, 3 Sep	26–27 Aug	27 Aug, 17 Sep	29–30 Jul	30 Jul–1 Aug	29 Jul–1 Aug	1–3 Sep	30 Sep–2 Oct
2015	25 Aug	25–26 Aug	26 Aug	26–27 Aug	4 Aug	5 Aug	4–5 Aug	2–3 Sep	28, 30 Sep

^a^The air dose rates are the averages and SD (*n* = 6 at KU1-H and KU1-Q, *n* = 20 at KU2-S and TB-H, *n* = 27 at KU1-S, *n* = 32 at TD-S, and *n* = 36 at OT-Q and OT-P) from the first observation date.

^b^The deposition of ^137^Cs at each plot was obtained from the 5th airborne monitoring survey data collected on 28 June 2012 and reported on the website “Extension Site of Distribution Map of Radiation Dose, etc.” prepared by MEXT, Japan^31^.

^c^For each component of every plot, the results for coniferous trees are shown on the left, and those for broad-leaved trees are shown on the right of each slash to denote the stand density, mean DBH, and aboveground biomass. The aboveground and belowground value are the ranges over the 4–5 years of the study.

As shown in studies on the Chernobyl incident, the distribution of ^137^Cs in forests changes with time, especially over the first 4–5 years after an accident. Aboveground ^137^Cs can migrate to underlying soils via litterfall, throughfall, and stemflow^8^, and the proportion of ^137^Cs in soils eventually increases. In addition, changes in ^137^Cs transfer to wood are of serious concern with regards to the future resumption of timber production. Since 2011, we have continued to monitor the abovementioned five plots in Fukushima to capture the changes in the ^137^Cs distribution in these forests. In addition, we established four new plots in 2011 or 2012, with continued monitoring: a hinoki cypress forest (KU1-H) and a broad-leaved forest (KU1-Q) located close to KU1-S, a Japanese cedar forest (KU2-S) located in the same village as KU1-S but possessing a smaller contamination level from that at KU1-S, and a hinoki cypress forest in Ishioka City (TB-H) located 160 km away from the FDNPP and possessing a similar contamination level as that at TD-S.

This paper reports our five-year (2011–2015) monitoring of the radiocesium distributions across nine forest plots containing variable tree species and different contamination levels. We sought to describe and quantify the overall changes in the ^137^Cs distributions within the forests based on our monitoring data, and we discuss the similarities and differences in these changes within forests among the plots.

## Results

### Radiocesium concentrations

**Figure 2:**
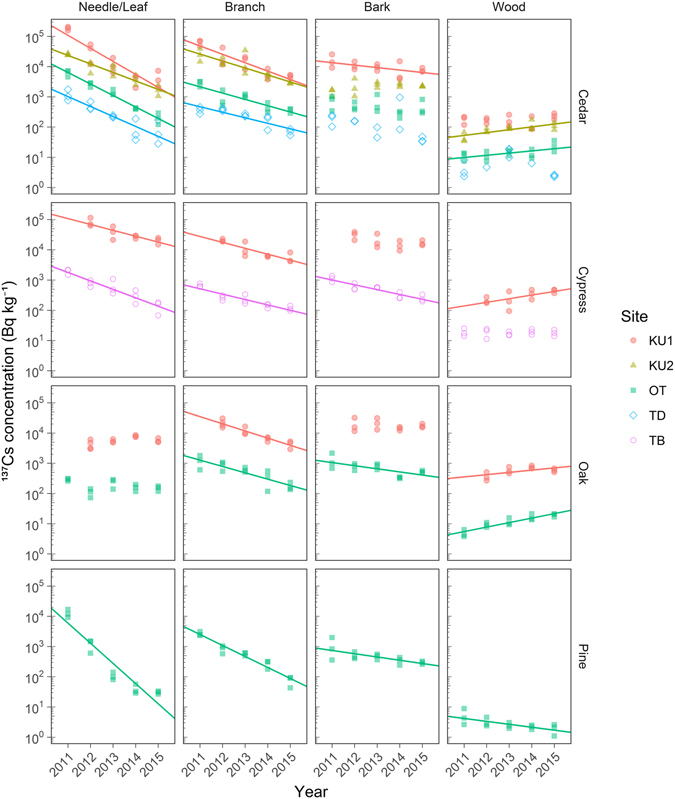
Time series of needle/leaf, branch, bark, and wood ^137^Cs concentrations at each study plot. For individual samples, log-transformed ^137^Cs concentrations above the detection limit were plotted against the sampling year. Maximum likelihood regression analysis was performed for data below the detection limit^38^. Significant trends between the log-transformed concentration and sampling year (linear regression analysis, *p* < 0.05) are denoted by solid lines. The sampling period was August–September in each year except at KU2-S in 2011 (November 2011) and TB-H in 2011 and 2012 (February 2012 and February 2013, respectively). The radiocesium concentration was decay corrected to September 1 of each sampling year.

**Figure 3:**
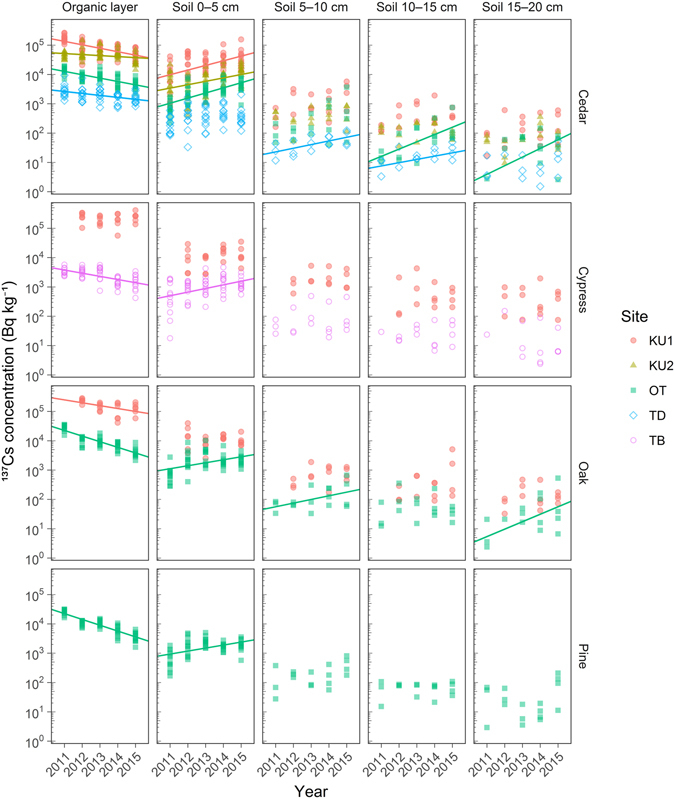
Time series of organic layer and mineral soil layer (0–5 cm, 5–10 cm, 10–15 cm, and 15–20 cm) ^137^Cs concentrations at each study plot. For individual samples, log-transformed ^137^Cs concentrations above the detection limit were plotted against the sampling year. Maximum likelihood regression analysis was performed for data below the detection limit^38^. Significant trends between the log-transformed concentration and sampling year (linear regression analysis, *p* < 0.05) are denoted by solid lines. The sampling period was August–September in each year except at KU2-S in 2011 (November 2011) and TB-H in 2011 and 2012 (February 2012 and February 2013, respectively). The radiocesium concentration was decay corrected to September 1 of each sampling year.

Figures 2 and 3 show the five-year changes in the ^137^Cs concentration (decay corrected to September 1 of each year and log-transformed) in needles/leaves, branches, bark, wood, the organic layer, and four mineral soil layers (0–5 cm, 5–10 cm, 10–15 cm, and 15–20 cm) for each study plot. Regression lines were drawn where the slopes were significantly positive or negative (*p* < 0.05). Note that the ^137^Cs concentrations did not necessarily change exponentially, particularly in the mineral soil layers, in which the ^137^Cs concentration first increased from 2011 to 2012 and then fluctuated (see cedar and oak plots in Fig. 3). The annual means and standard deviations (SDs) of the ^137^Cs (and ^134^Cs) concentration in each component are shown online in Supplementary Table S1 of the supplementary information section.

Overall, the ^137^Cs concentrations in each component were positively correlated with the total level of deposition; ^137^Cs concentrations were highest at KU1-S (630 kBq m^**−**2^ in ^137^Cs deposition based on aerial survey) and lowest at TD-S (10 kBq m^**−**2^ in ^137^Cs deposition) (Figs 2 and 3 and Table 1). Needles, branches, bark, the organic layer, and the shallowest mineral soil layer (0–5 cm) (directly exposed to air) contained higher concentrations than wood and deeper mineral soil layers (not exposed to air). The ^137^Cs concentrations in needles, branches, and the organic layer showed clear decreasing trends, whereas those in wood and the shallowest soil layer (0–5 cm) showed increasing trends at the majority of plots. To remove the effect of radioactive decay on temporal ^137^Cs changes, we also conducted regression analysis using ^137^Cs data that was decay corrected to March 11, 2011 (Supplementary Figs S1 and S2). The decay correction to March 11, 2011, resulted in insignificant decreasing trends in bark at KU1-S and the organic layer at KU2-S and significant increasing trends in leaves at KU1-Q, bark at KU2-S, mineral soil (0–5 cm) at TD-S, and mineral soil (5–10 cm) at OT-S and OT-P. Most detected trends were not affected by the correction to March 11, 2011, suggesting that the trends observed in our study primarily resulted from radiocesium transfer.

The concentration of ^137^Cs in needles decreased exponentially at all coniferous plots (Fig. 2). The effective ecological half-lives of ^137^Cs in the needles were 0.70 (KU1-S), 1.07 (KU2-S), 0.79 (OT-S), 0.91 (TD-S), 1.55 (KU1-H), 1.08 (TB-H), and 0.45 (OT-P) yr, being shortest in Japanese red pine needles at OT-P and longest in hinoki cypress needles at KU1-H. The ^137^Cs concentration in the Japanese red pine needles in 2015 was to 0.2% of the initial concentration (2011), whereas those in the Japanese cedar and hinoki cypress needles in 2015 were 2–7% of the initial respective concentrations. The ^137^Cs concentration in oak leaves at the first monitoring time point (2011 for OT and 2012 for KU1) was approximately one-tenth of that in coniferous needles sampled at the adjacent plot in the same site and did not change over the monitoring period (5 years for OT and 4 years for KU1). Consequently, as of 2015, the ^137^Cs concentration in the oak leaves was the same order of magnitude as that in the coniferous needles at KU1 and OT. The concentration of ^137^Cs in branches exponentially decreased at all the study plots (Fig. 2). The effective ecological half-lives of ^137^Cs in the branches were 1.09 (KU1-S), 1.30 (KU2-S), 1.44 (OT-S), 1.65 (TD-S), 1.54 (KU1-H), 1.69 (TB-H), 1.27 (KU1-Q), 1.44 (OT-Q), and 0.83 (OT-P) yr. Similar to the needles, the ^137^Cs concentration in the branches of Japanese red pine decreased the fastest among the studied species. The concentration of ^137^Cs in bark decreased by 53–78% over the 4–5 years at four plots (KU1-S, TB-H, OT-Q, and OT-P) and did not show clear temporal variations at the other five plots (Fig. 2). The ^137^Cs concentration increased by 37–75% in konara oak wood (KU1-Q and OT-Q) during the 4–5 years and decreased by 63% in Japanese red pine wood (OT-P) over the 5 years (Fig. 2). The concentration of ^137^Cs in Japanese cedar and hinoki cypress woods showed increasing trends at half of the plots (Fig. 2).

In the organic layer, the ^137^Cs concentration decreased by 28–86% over the 4–5 years at all the plots except KU1-H (Fig. 3). Exponential decreasing trends were clearly observed at some plots (KU1-S, OT-S, TD-S, TB-H, OT-Q, and OT-P). In the surface mineral soil layer (0–5 cm), the concentration of ^137^Cs increased by 43–82% over the five years at six (KU1-S, KU2-S, OT-S, TB-H, OT-Q, and OT-P) of the nine plots (Fig. 3). Regarding annual changes, the ^137^Cs concentration in the surface mineral soil layer at some plots increased rapidly from 2011 to 2012 and then levelled off or fluctuated in later years (Soil 0–5 cm in Fig. 3). In deeper mineral soil layers (below 5 cm), the concentrations of ^137^Cs did not show clear temporal trends at almost all plots, with the exception of TD-S (5–10 cm and 10–15 cm), OT-S (10–15 cm and 15–20 cm), and OT-Q (5–10 cm and 15–20 cm), where increasing trends were observed (Fig. 3). In particular, after applying the decay correction to March 11, increasing trends were observed in deeper soil layers at some plots, which suggests that ^137^Cs continued to migrate very slowly to deeper soil layers during the five years, although this migration was very minor compared to the rapid migration during the first half year after the fallout.

## Radiocesium inventories

**Figure 4:**
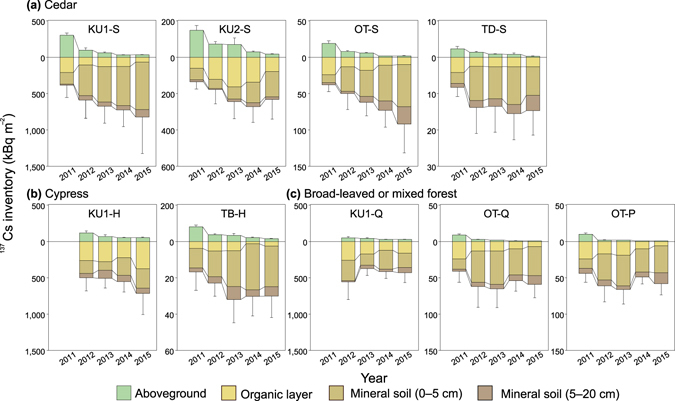
Time series of the ^137^Cs inventories in the aboveground compartments, organic layer, and mineral soil layers at each study plot. Error bars denote the SDs of aboveground and belowground ^137^Cs inventories.

Figure 4 shows temporal changes in the ^137^Cs inventory in the aboveground compartments (needles/leaves, branches, bark, and wood), organic layer, and mineral soil layers at each study plot. The ^137^Cs (and ^134^Cs) inventory of each component is shown online in Supplementary Tables S2 and S3 of the supplementary information section.

The aboveground compartments retained the highest percentage of ^137^Cs in 2011 (18–52% of the total ^137^Cs inventory) and then decreased in subsequent years in each plot (to 1.0–7.0% in 2015; Supplementary Tables S2 and S3). This rapid decline in the ^137^Cs inventory of aboveground compartments was mainly due to decreases in the needle and branch inventories, which retained more than 80% of the ^137^Cs initially deposited in the aboveground compartments in 2011 (Supplementary Tables S2 and S3). The ^137^Cs inventory in wood accounted for less than 1% of the total ^137^Cs inventory at all the plots except TB-H (<2%) in 2015 (Supplementary Tables S2 and S3). The proportion of the aboveground ^137^Cs inventory was higher at sites KU1, KU2, and TB (4–7% in 2015) than at sites OT and TD (1–2%) (Supplementary Tables S2 and S3). The decrease in the aboveground ^137^Cs inventory was most significant from 2011 to 2012 (Fig. 4). In particular, the ^137^Cs inventory in the aboveground compartments decreased to 4% in 2012 at the mixed forest plots (OT-Q and OT-P), representing a more significant decrease than the decrease to 9–29% at the Japanese cedar and hinoki cypress plots (Supplementary Tables S2 and S3). The faster rate of decline observed at the mixed forest plots was mainly attributed to the significant decline in the ^137^Cs concentration in the Japanese red pine needles (Fig. 2). By contrast, the ^137^Cs inventory in the aboveground compartments remained approximately stable at the broad-leaved forest plot (KU1-Q), where the proportion of the ^137^Cs inventory in leaves remained consistently low (0.2–0.5% of the total ^137^Cs inventory; Supplementary Tables S2 and S3). Consequently, the ^137^Cs percentage in the aboveground compartments was comparable after 2013 among the different forest types at KU1 (Fig. 4).

The ^137^Cs inventory in the organic layer was highest in 2011 (16–49% of the total ^137^Cs inventory) and then decreased over time at most plots (to 8–35% in 2015 at KU1-S, OT-S, TD-S, TB-H, KU1-Q, OT-Q, and OT-P; Fig. 4 and Supplementary Tables S2 and S3). However, the ^137^Cs inventory in the organic layer increased from 2011 to 2013 and then decreased at KU2-S (Fig. 4), where the ^137^Cs proportion in the aboveground compartments was highest among the plots in 2011 (52%; Supplementary Tables S2 and S3). At the Kawauchi site (KU1-H, KU1-Q, and KU2-S), higher ^137^Cs levels persisted in the organic layer (31–49% in 2015), in contrast to those at the other sites (8–17%) except KU1-S (8%) (Supplementary Tables S2 and S3).

Corresponding to the ^137^Cs decline in the aboveground compartments and organic layers, the annual ^137^Cs inventory in the mineral soil layers was lowest in 2011 (25–39%) and then drastically increased from 2011 to 2012 (64–75% in 2012) at KU1-S, OT-S, TD-S, OT-Q, and OT-P (Fig. 4 and Supplementary Tables S2 and S3). Over the five years studied, mineral soils became the largest ^137^Cs reservoirs within the forests at these plots (81–89% in 2015) (Supplementary Tables S2 and S3). To a depth of 20 cm, the majority of the ^137^Cs in mineral soils was stored in the 0–5 cm layer at all plots (65–91% of the ^137^Cs inventory in the mineral soil layers in 2015).

## Discussion

### Aboveground ^137^Cs

Our five-year monitoring data collected at plots with different contamination levels revealed the same exponential decreases in the ^137^Cs concentration in needles and branches at all coniferous plots (Fig. 2). Previous studies have shown that throughfall and litterfall sampled in various forest stands affected by Fukushima fallout are highly contaminated with radiocesium^9–11^ and that throughfall contributions to ^137^Cs transfer from aboveground to belowground were more significant than those of defoliation in the very early phase after the incident and then rapidly decreased^9, 12^. Therefore, the observed decreases in ^137^Cs in needles and branches were probably first mainly driven by rain; thereafter, defoliation played a major role in the decreases, and these processes similarly occurred at all plots overall.

Among the coniferous species, the effective ecological half-life of ^137^Cs in needles was the shortest for Japanese red pine compared to those of Japanese cedar and hinoki cypress. We inferred that Japanese red pine trees defoliated their needles that were initially contaminated at the time of Fukushima accident more rapidly than the other coniferous tree species because Japanese red pine trees have a shorter leaf longevity (2–3 years) than Japanese cedar and hinoki cypress (4–6 years)^13, 14^. For konara oak, because significant deposition resulting from the Fukushima accident occurred at a stage prior to leaf flushing, the deciduous leaves of konara oak were much less contaminated than other evergreen needles in the summer of 2011^7^. In addition, the ^137^Cs concentration in the deciduous leaves did not change significantly over the five years, whereas the concentrations in other species exponentially decreased. Interestingly, as a result, the ^137^Cs concentration in deciduous leaves reached levels comparable to that in evergreen needles at the adjacent plot in the same site (KU1 and OT) after five years (Fig. 2).

Tree bark was less contaminated in the summer of 2011 than needles and branches at many plots (Fig. 2) likely because needles and branches were more exposed to radiocesium deposition than bark and because needles and branches are morphologically effective at intercepting deposition. However, our five-year monitoring study revealed that the ^137^Cs concentration in bark did not change considerably over the observation period at many plots. Bark mainly consists of inner bark (phloem) and outer bark. The outer bark, which was exposed to radiocesium deposition, would retain a considerable portion of radiocesium in the early phase^15^. Therefore, the longer residence time of ^137^Cs in the bark than in the needles and branches implies that both the turnover and wash-off rates in the outer bark are low compared to those in the needles and branches. Eventually, the concentration and inventory of bark ^137^Cs reached comparable levels to those of needle and branch ^137^Cs in 2015 (Fig. 2 and Supplementary Tables S2 and S3).

These unique behaviour of bark ^137^Cs have been reported in Chernobyl studies: The bark of an oak tree species (*Q. petraea*) in Bulgaria was found to be 2–4 times more contaminated with ^137^Cs than its leaves and branches 22 years after the Chernobyl accident^16^. In the 40-mm-thick bark of an oak tree species (*Q. robur*) in Romania, the outer 8-mm layer was found to be the most contaminated with ^137^Cs 27 years after the accident^17^. Alternatively, the ^137^Cs concentration in inner bark was higher than that in outer bark in Scots pines 12 years after the accident^18^. These differences in outer and inner bark ^137^Cs appear to be dependent on the species and/or environment, and careful follow-up research on bark contamination is therefore of particular importance to forest management.

### Belowground ^137^Cs

The ^137^Cs concentration in the organic layers decreased exponentially with time at some plots, whereas that in the surface mineral soil layer increased (Fig. 3). These results indicate that ^137^Cs was transferred from the organic layer to the mineral soil layer, which was likely caused by washout by rain and litter decomposition^19^ over the five years. As an exception, the ^137^Cs inventory in the organic layer increased from 2011 to 2013 at KU2-S, where the ^137^Cs proportion in the aboveground compartments was highest over the five years compared to that at the other plots (Fig. 4 and Supplementary Tables S2 and S3). As the amount of ^137^Cs retained by needles and bark was relatively high at KU2-S, this high interception resulted in the delayed migration of ^137^Cs from the aboveground compartments to the organic layer. This delayed migration explains the increasing ^137^Cs inventories from 2011 to 2013 and the decreasing inventories from 2013 to 2015 in the organic layer. Previous studies have reported that the thickness and volume of the organic (litter) layer affect the ^137^Cs retention capacity of the organic layer^20, 21^. We did not detect high retention within the thick organic layer (e.g., 1.4–2.8 cm at KU2-S and 1.6–2.1 cm at OT-S) (Table 1 and Fig. 4). However, the proportions of ^137^Cs retained in the organic layer to belowground compartments (organic layer and mineral soil) were relatively high at KU1-H, KU1-Q, and KU2-S in 2015. At these plots, the ^137^Cs proportions in aboveground compartments were high (Supplementary Tables S2 and S3), which may suggest that the amount of ^137^Cs in the organic layer was more closely controlled by inputs from aboveground trees rather than by the ^137^Cs retention capacity of the organic layer during the initial phase of radiocesium cycling after the fallout.

In the mineral soil layers, the most ^137^Cs was retained in the shallowest layer, as shown in previous reports^21–23^. The retention rate of the surface layer (the ratio of the ^137^Cs inventory in the 0–5 cm soil layer to that in all soil layers) did not show any temporal trends at any of the plots (data not shown). Matsunaga *et al*. (2013)^24^ reported that the vertical distribution of ^137^Cs in mineral soils did not change after the rainy season in the first year following the FDNPP accident. The ^137^Cs flux through the mineral soil layers decreased in the second year relative to level in the first year^20, 25^. Previous studies on the Chernobyl incident have reported that the ^137^Cs level in mineral soil decreased with increasing depth and that the distribution of ^137^Cs in mineral soils did not change drastically over time^26^. These findings suggest that the ^137^Cs distribution in the mineral soil layer was rapidly established within a half year after the fallout at Fukushima and that the mobility of ^137^Cs in the mineral soil layer quickly decreased, although ^137^Cs continues to migrate to deeper soil layers very slowly at some plots.

### ^137^Cs in wood

Time series variations of the ^137^Cs concentration in wood differed between tree species: The concentrations in Japanese red pine decreased, whereas those in konara oak increased (Fig. 2). Japanese cedar and hinoki cypress showed increasing trends or did not show significant trends (Fig. 2). Regarding the Chernobyl accident, oak trees exhibited stronger ^137^Cs absorption capacities than pine trees^20, 27^, in accordance with our results. Recent Fukushima studies have noted the relative importance of the foliar uptake of radiocesium and its translocation in the initial phases following deposition^28–30^. The radiocesium concentrations in internal tree tissues should be affected by many factors such as foliar uptake just after an accident, subsequent root uptake, dilution due to tree growth, radioactive decay, and defoliation. The relative biomass increment in woods was low at OT-Q (0.3% yr^**−**1^) and OT-P (0.8% yr^**−**1^) compared to that at other coniferous plots (2–9% yr^−1^; Supplementary Table S4). Therefore, the dilution of the ^137^Cs concentration in wood due to tree growth was almost negligibly small in the Japanese red pines and konara oaks in the present study. In addition, in the present study, the radioactive decay of ^137^Cs (**−**2.3% yr^**−**1^) did not affect the temporal trends in the ^137^Cs concentration in wood because the significance of the slope of the regression line did not change after decay correction to March 11, 2011 (Supplementary Fig. S1). Foliar uptake of ^137^Cs would have had little influence on the temporal trends in the ^137^Cs concentration in konara oak wood because the leaves had not been flushed at the time of the accident. Thus, the increasing trends in the ^137^Cs concentration in konara oak wood suggest that the ^137^Cs inflow exceeded the outflow due to defoliation during the five years after the accident. By contrast, the decreasing trend in Japanese red pine wood indicates that the ^137^Cs inflow was smaller than the outflow. The ^137^Cs inflow due to root uptake, however, can be affected by soil conditions such as moisture^20^ and potassium and ammonium contents^26^, and the outflow due to defoliation might be affected by the vitality and age of a tree. The different temporal trends observed in the Japanese cedar and hinoki cypress among the sites should be evaluated in further studies that include such pedological and physiological details.

## Concluding remarks

In this paper, we reported temporal radiocesium changes after the FDNPP accident based on annual monitoring data of the radiocesium distributions in Fukushima forests of various types and with varying contamination levels from 2011 to 2015. Our multi-site, multi-species monitoring results revealed that the pattern of temporal radiocesium changes in the 9 forest plots was similar overall; however, changes in ^137^Cs in needles/leaves and wood differed among the tree species. These suggest that our data are very useful for understanding and quantifying radiocesium migration in various forests in Fukushima and for developing a predictive model. The distributions of radiocesium in forests will continue to change until steady state is reached, and further monitoring will undoubtedly be required in the future.

## Methods

### Sites

The study was conducted at five sites with different radiocesium deposition levels: site KU1 on Mt. Otsupe in Kawauchi Village (26–27 km from the FDNPP, 470–630 kBq m^**−**2^ of ^137^Cs deposition as of June 2012, as evaluated by airborne monitoring^31^), site KU2 at Kanayama in Kawauchi Village (28 km, 160 kBq m^**−**2^), site OT in Otama Village (66 km, 41–44 kBq m^**−**2^), and site TD in Tadami Town (134 km, 10 kBq m^**−**2^) in Fukushima Prefecture, Japan, as well as site TB on Mt. Tsukuba in Ishioka City (160 km, 33 kBq m^**−**2^) in Ibaraki Prefecture, Japan (Table 1 and Fig. 1). From August–September 2011, study plots at KU1-S, OT-S, and TD-S were established in Japanese cedar (*Cryptomeria japonica*; common name: sugi) plantations. Plot OT-Q was located in a secondary forest where deciduous oak (*Quercus serrata*; common name: konara) is as abundant as pine, and plot OT-P was located in a secondary forest where Japanese red pine (*Pinus densiflora*; common name: akamatsu) dominates, along with some broad-leaved tree species^7^. In addition, KU2-S was established in a Japanese cedar plantation in November 2011; TB-H and KU1-H were established in a hinoki cypress (*Chamaecyparis obtusa*; common name: hinoki) plantation in February 2012 and August 2012, respectively; and KU1-Q was established in a secondary forest of DBL trees in August 2012. The topographic features, plot areas, initial deposition levels, stand ages as of 2011, stand densities and diameters at breast height (DBH), aboveground biomass levels, and forest floor surface densities for each study plot are shown in Table 1. Steep slopes were found at KU1-S, OT-S, TD-S, and TB-H. Soils were classified as Andosols or brown forest soils (KU1-S, OT-S, OT-Q, and OT-P) and as Inceptisols or brown forest soils (TD-S)^23, 32^. The dry mass of the organic layer (kg m^**−**2^) was highest at KU2-S. The annual mean temperature was 10.7 °C, 15.0 °C, 10.2 °C, and 14.4 °C, and rainfall levels reached 1,574 mm, 1,176 mm, 2,615 mm, and 1,435 mm in 2011–2015 at nearby meteorological stations of KU, OT, TD, and TB (Supplementary Table S5) (Japan Meteorological Agency)^33^.

### Field measurements and sampling

Field measurements and sampling of needles/leaves, branches, bark, wood, litter, and mineral soils were basically conducted from August to September of each year (Figs 2 and 3). At each study plot, tree monitoring had been carried out annually to determine the species, diameter growth, and mortality and to recruit all individual trees with a DBH of >10 cm. Tree heights were estimated from the observed relationship between tree height (approximately 20 randomly selected trees per plot) and DBH. The biomass values of needles/leaves and branches were estimated based on DBH allometric equations for dominant species^34^. Biomass values of bark and wood were estimated from the total stem volume, which was calculated from the DBH and tree height^34^ and from the mean volume ratio and specific density of wood and bark samples^35^.

Three trees of differing diameters (small, medium, and large) were felled near the study plot of each stand every year: Japanese cedar at KU1-S, KU2-S, OT-S, and TD-S; konara oak and Japanese red pine at OT-Q and OT-P; konara oak near KU1-Q; and hinoki cypress near KU1-H and TB-H. Needles/leaves and branches were sampled from different parts of each tree crown. These samples were homogenized and then oven-dried for 2–3 days at 75 °C. Bark and debarked wood disks were sampled from stems at approximately breast height, and disks were divided into sapwood and heartwood samples. Bark and wood samples taken in 2011 were oven-dried for over 2 days at 105 °C. From 2012, to facilitate drying, bark and wood samples were oven-dried for 2–3 days at 75 °C, and the dry masses were converted at 105 °C using mean conversion factors (0.98 for bark and 0.99 for wood), which were experimentally determined for the four species.

Organic and mineral soil layer samples were collected at four points around three trees in 2011. After 2012, sampling subplots were established at 12 points in KU1-S, KU2-S, OT-S, OT-Q, OT-P, TD-S, and TB-H and at 6 points in KU1-H and KU1-Q, where the stand areas were relatively small. Samples of the organic layer accumulated on the forest floor were collected from a 25 × 25-cm quadrat. Soil layers were sampled at 5-cm intervals from 0 cm to a depth of 20 cm using a 475-mL cylinder (95-cm^2^ cross-sectional area × 5-cm depth). Details on the sampling method used are reported in Ikeda *et al*.^36^. Samples of the organic and surface soil layers (0–5 cm depth) were collected from each subplot. Deeper soil layers (5–10, 10–15, and 15–20 cm) below the organic and surface soil layers were sampled at 4 or 3 points in each plot. The organic and mineral soil layer samples were air-dried and then oven-dried for 24 h at 75 °C and 105 °C, respectively.

### Radiocesium analysis

The concentrations of ^137^Cs and ^134^Cs in most samples from 2011 were determined using a gamma-ray spectrometer (GC2020-7500SL-2002CSL, Canberra, Meriden, USA). Further details on this method are reported in Kuroda *et al*.^37^, Fujii *et al*.^23^, and Komatsu *et al*.^7^. The ^134^Cs and ^137^Cs concentrations in all samples from 2012–2015 were determined using HPGe coaxial and P-type reverse coaxial detector systems (GEM20-70, GEM40P4-76, GEM-FX7025P4-ST, and GWL-120-15-LB-AWT, ORTEC, Oak Ridge, USA) and spectral analysis software (DS-P1001 Gamma Station, Seiko EG&G, Tokyo, Japan) in the Forestry and Forest Products Research Institute (FFPRI) laboratory. Measurement times were set to 1,800–86,400 s to obtain ^137^Cs concentrations with relative errors of <10%. When concentrations fell below the detection limit, the mean value and SD were determined via maximum likelihood estimation^38^. This procedure for processing data below the detection limit was also applied in the regression analysis. The radiocesium concentration was decay corrected to September 1 of each sampling year. The radiocesium concentration in wood was derived by weighting the mean radiocesium concentration of sapwood and heartwood by the biomass of sapwood and heartwood for each sample. The radiocesium inventory (Bq m^**−**2^) of each component was calculated by multiplying the mean radiocesium concentration per unit of dry mass (Bq kg^**−**1^) by the dry mass of each unit area (kg m^**−**2^). Further information on the stand-level radiocesium inventory calculations is included in Komatsu *et al*.^7^. We confirmed that the ^134^Cs/^137^Cs ratios of the samples were very stable after considering their half-lives; hence, we hereafter focus only on the ^137^Cs results in this paper (see the supplementary information section for ^134^Cs data).

The effective ecological half-lives (*T* ^eff^) denoting the reduction of ^137^Cs activity concentration in the leaves and branches were calculated with the follow equation:

1 $$C(t)=C(0)\cdot {e}^{-\frac{\mathrm{ln}2}{{T}^{eff}}t}$$

where *C*(0) is the initial concentration (Bq kg^**−**1^), sampled in 2011 or 2012 according to the sampled plots (Table 1) and *C*(*t*) is the ^137^Cs concentration (Bq kg^**−**1^) after time *t* (yr). Both *C*(*t*) and *C*(0) are decay corrected to September 1 of each sampling year. Note that the effective ecological half-life involves both the physical and ecological half-lives.

## Electronic supplementary material

Supplementary Figures

Supplementary Tables
