# Supplementary material for: Temporal changes in the radiocesium distribution in forests over the five years after the Fukushima Daiichi Nuclear Power Plant accident
Source: Sci Rep. 2017 Aug 15;7:8179. doi: 10.1038/s41598-017-08261-x (PMC5557944; doi:10.1038/s41598-017-08261-x)
Supplement: Supplementary file 1 — Supplementary Figures [file 41598_2017_8261_MOESM1_ESM.pdf]

Temporal changes in the radiocesium distribution in forests over the five years after the Fukushima Daiichi Nuclear Power Plant accident

Naohiro Imamura, Masabumi Komatsu, Shinta Ohashi, Shoji Hashimoto, Takuya Kajimoto, Shinji Kaneko, Tsutomu Takano

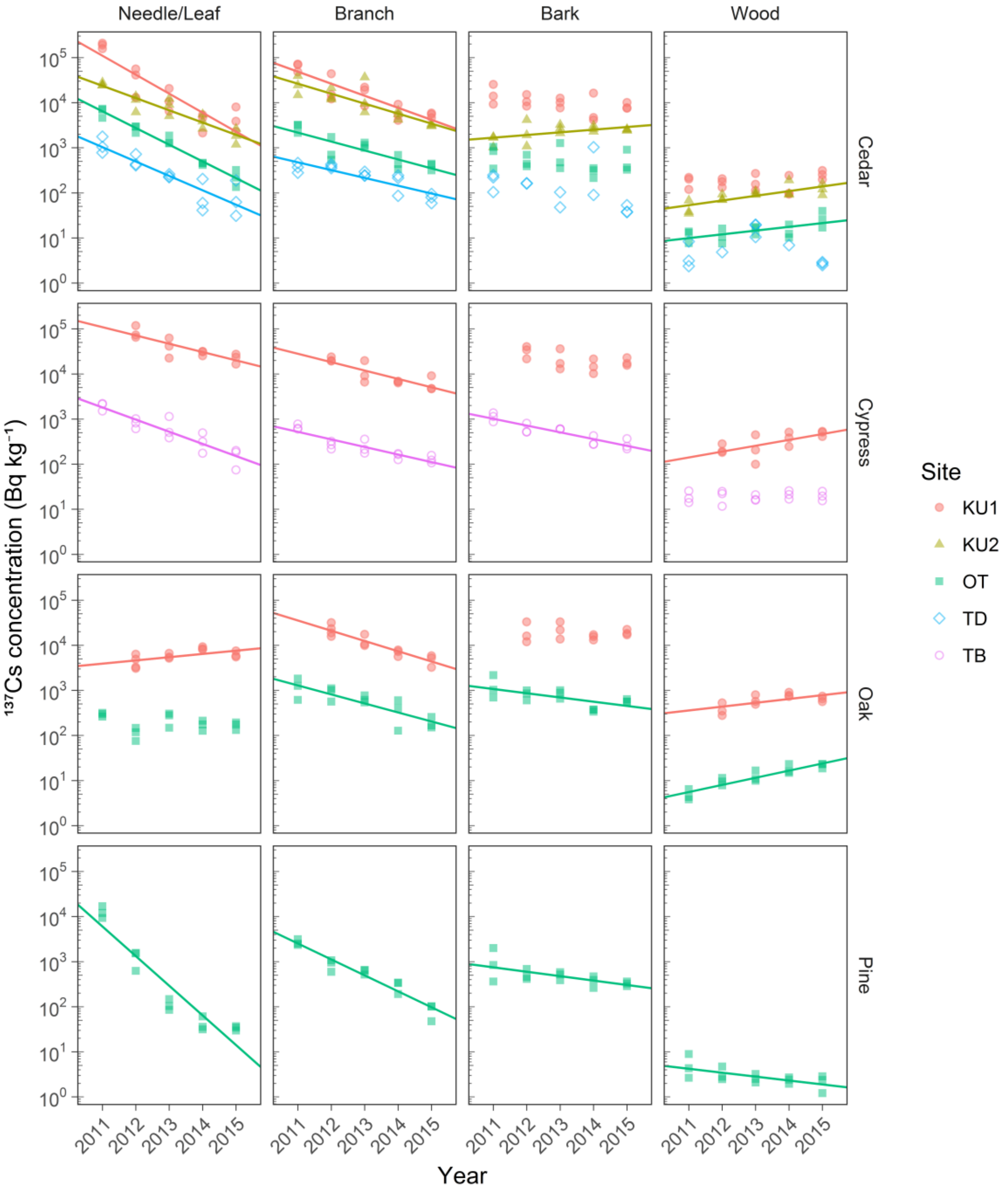

Supplementary Figure S1. Time series of the needle/leaf, branch, bark, and wood ^{137}Cs concentrations at each study plot. For individual samples, log-transformed ^{137}Cs concentrations above the detection limit were plotted against the sampling year. Maximum likelihood regression analysis was performed for data below the detection limit[37]. Significant trends between the log-transformed concentration and sampling year (linear regression analysis, $p < 0.05$) are denoted by solid lines. The sampling period was August–September in each year except at KU2-S in 2011 (November 2011) and TB-H in 2011 and 2012 (February 2012 and February 2013, respectively). The radiocesium concentration was decay corrected to March 11, 2011.

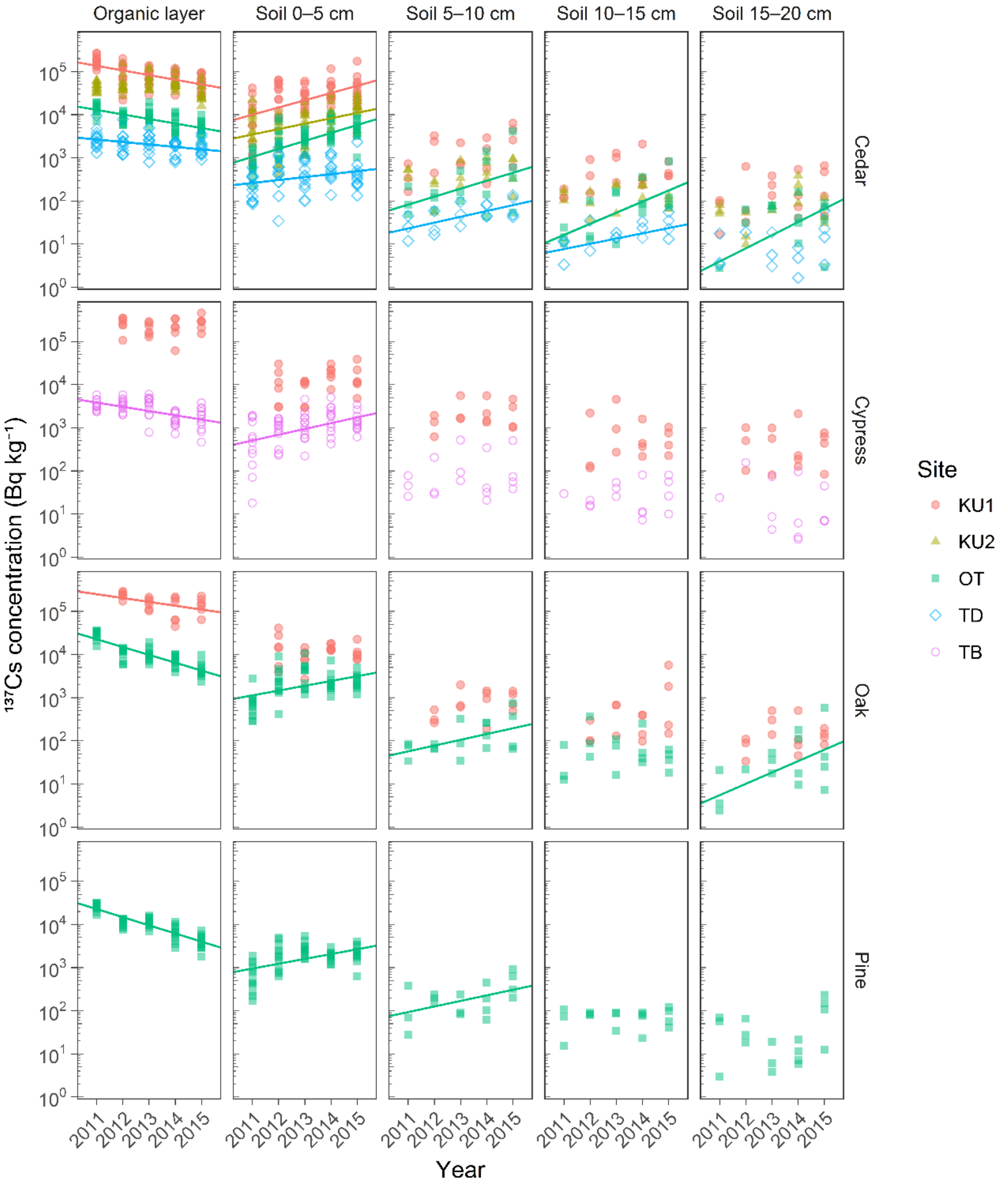

Supplementary Figure S2. Time series of the organic layer and mineral soil layer (0–5 cm, 5–10 cm, 10–15 cm, and 15–20 cm) ^{137}Cs concentrations at each study plot. For individual samples, log-transformed ^{137}Cs concentrations above the detection limit were plotted against the sampling year. Maximum likelihood regression analysis was performed for data below the detection limit[37]. Significant trends between the log-transformed concentration and sampling year (linear regression analysis, $p < 0.05$) are denoted by solid lines. The sampling period was August–September in each year except at KU2-S in 2011 (November 2011) and TB-H in 2011 and 2012 (February 2012 and February 2013, respectively). The radiocesium concentration was decay corrected to March 11, 2011.
